# Pulse Diagnosis Signals Analysis of Fatty Liver Disease and Cirrhosis Patients by Using Machine Learning

**DOI:** 10.1155/2015/859192

**Published:** 2015-11-28

**Authors:** Wang Nanyue, Yu Youhua, Huang Dawei, Xu Bin, Liu Jia, Li Tongda, Xue Liyuan, Shan Zengyu, Chen Yanping, Wang Jia

**Affiliations:** ^1^Experimental Research Center, China Academy of Chinese Medical Sciences, Beijing 100700, China; ^2^Department of Computer Science and Technology, Tsinghua University, Beijing 100084, China; ^3^GULOU Hospital of TCM of Beijing, Beijing 100009, China; ^4^Tai Yang Gong Health Care Center, Chaoyang District, Beijing 100102, China; ^5^Shi Xuemin Academician Office, First Teaching Hospital of Tianjin University of Traditional Chinese Medicine, Tianjin 300193, China

## Abstract

*Objective*. To compare the signals of pulse diagnosis of fatty liver disease (FLD) patients and cirrhosis patients.* Methods*. After collecting the pulse waves of patients with fatty liver disease, cirrhosis patients, and healthy volunteers, we do pretreatment and parameters extracting based on harmonic fitting, modeling, and identification by unsupervised learning Principal Component Analysis (PCA) and supervised learning Least squares Regression (LS) and Least Absolute Shrinkage and Selection Operator (LASSO) with cross-validation step by step for analysis.* Results*. There is significant difference between the pulse diagnosis signals of healthy volunteers and patients with FLD and cirrhosis, and the result was confirmed by 3 analysis methods. The identification accuracy of the 1st principal component is about 75% without any classification formation by PCA, and supervised learning's accuracy (LS and LASSO) was even more than 93% when 7 parameters were used and was 84% when only 2 parameters were used.* Conclusion*. The method we built in this study based on the combination of unsupervised learning PCA and supervised learning LS and LASSO might offer some confidence for the realization of computer-aided diagnosis by pulse diagnosis in TCM. In addition, this study might offer some important evidence for the science of pulse diagnosis in TCM clinical diagnosis.

## 1. Introduction

Pulse diagnosis had played an important role in clinical diagnosis and therapeutic evaluation of TCM for several thousand years. Modern researches of pulse diagnosis based on the modern technology, such as the signal analysis, are very important for the development of TCM.

Machine learning builds empirical models on data for analysis and forecasting, which has recently been used for TCM data analysis [1], especially for the diagnosis data of TCM. Some research about modeling and symptom selection for multilabel data in the inquiry diagnosis of coronary heart disease (CHD) [1, 2] and multiclass support vector machines in lip diagnosis [3] offered some available new methods in modern research of TCM. Pulse diagnosis is more difficult in standardization researches. Our work team had done some work about it, including data analysis for pregnant women, animals with High Blood Pressure and Heart Failure, rats after nephrectomy, and patients with CHD, HBP, and so forth [4–7]. The results are encouraging.

Supervised learning and unsupervised learning are the primary methods of machine learning. In the research of pulse diagnosis in TCM, it is very difficult to collect large number of samples with high quality. Accordingly, in this study, we combined unsupervised (PCA) with supervised methods (LS and LASSO) to analyze the signals collected from patients with different diseases and healthy volunteers for cross-reference to achieve reliable results in identification by signals of pulse diagnosis in TCM.

Fatty liver disease (FLD) and cirrhosis are both common liver diseases in clinic with high incidence. FLD is generally described as the build-up of fat in the liver cells. The prevalence of nonalcoholic fatty liver disease (NAFLD) ranges from 9 to 36.9% of the population in different parts of the world [8–10]; even in army, the incidence of nonalcoholic fatty liver disease is about 17.1% in navy flight crew and submariners [11].

Cirrhosis is a result of advanced liver disease. It is characterized by replacement of liver tissue by fibrosis and regenerative nodules. Cirrhosis is most commonly caused by alcoholism, hepatitis B and hepatitis C, and fatty liver disease. Cirrhosis is a leading cause of death in the world. In Europe, 95,609 males and 53,123 females died of cirrhosis in 2002, with large differences in age adjusted death rates among the different European geographical areas [12]. Complications such as ascites, esophageal variceal bleeding, hepatic encephalopathy, and hepatorenal syndrome are the main cause of death in this kind of disease.

Traditional Chinese Medicine plays an important role in the treatment of the two diseases, and pulse diagnosis can help clinic doctors during the diagnosis and the treatment, including prescription and evaluation. Experienced doctors can feel the difference between patients and healthy people just by pulse feeling; someone can even separate cirrhosis patients from FLD patients, but we get no evidence.

In this study, we would like to analyze the pulse signals collected by pulse-collecting instrument, and 3 groups of people are collected: healthy volunteers, patients with FLD, and patients with cirrhosis. Supervised learning and unsupervised learning are used in this study, and the results are encouraging.

## 2. Objects

We collected the pulse waves of 100 healthy volunteers in the graduated students' institute of China Academy of Chinese Medical Sciences and Tsinghua University. 50 patients with FLD were collected in China Academy of Chinese Medical Sciences and 50 patients with cirrhosis were collected in Guang'anmen Hospital of China Academy of Chinese Medical Sciences from December 2012 to July 2013. All the volunteers and patients were asked to fill the questionnaires. According to the quality of the signals and the integrity of their information we chose 98 cases from the healthy volunteers, 38 cases from the patients with FLD, and 27 cases from the patients with cirrhosis.

## 3. Methods

### 3.1. Signal Collection

**Figure 1:**
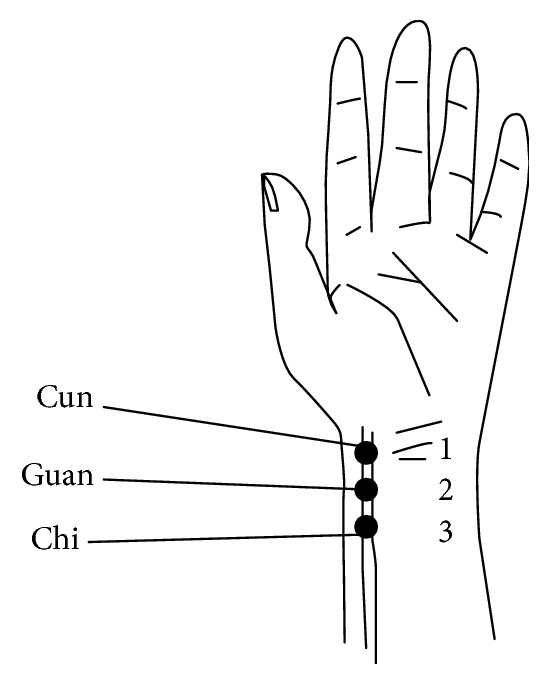
Places of “cun,” “guan,” and “chi” in the left hand.

Volunteers were asked to sit and keep silence to adjust their breath for 15 min firstly; then we collect the pulse waves in 3 places of both left and right sides of the radial artery called “cun,” “guan,” and “chi” in TCM (Figure 1 shows the details) for 40 s by the instrument called “Collection and Analysis System of Pulse Diagnosis Signals in TCM (patent number of pulse-collecting instrument: 200810225717.0).

This study had been demonstrated by the ethics committee of Experimental Research Center of China Academy of Chinese Medical Sciences, and each of the patients and volunteers had read and signed the informed consent.

### 3.2. Pretreatment and Parameters Extracting Based on the Harmonic Fitting

#### 3.2.1. Pretreatment

**Figure 2:**
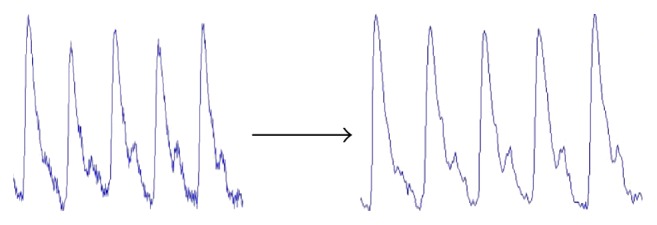
Pretreatment result: the figure on the left side is the original signal, and the figure on the right side is the signal after pretreatment; the main characters remain unchanged but the noise effectively reduced.

**Figure 3:**
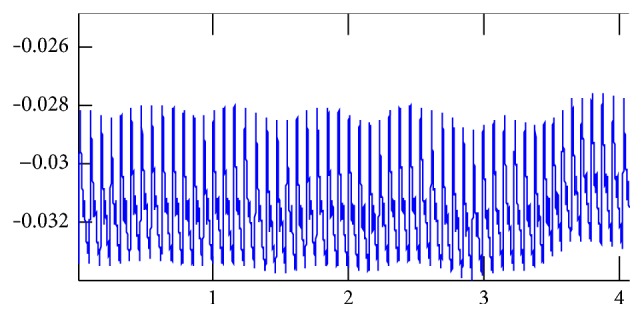
Original signals: the coordinate values of the start point of the signals regularly change because of the human breath.

**Figure 4:**
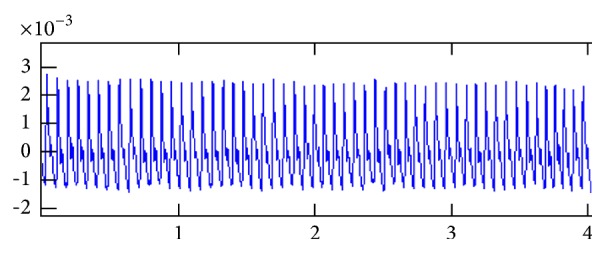
Pretreated signals: the coordinate values of the start point of the signals had been adjusted to zero, without any influence on the amplitude and other characters.

We do pretreatment [4] for the signals by the database administration and analysis system of pulse diagnosis signals in TCM. The first step is to filter the frequencies out of the range from 0.5 Hz to 48 Hz by DCT and then IDCT to recover the pulse waves without those components. The pretreatment results are shown in Figures 2, 3, and 4.

#### 3.2.2. Periodic Division

We do periodic division for the pulse waves by the speed and acceleration of signals' intension and changes. Regarding the fact that the periods of human pulse are not exactly the same no matter the status, the lengths and the figures of each cycle are also not completely similar.

#### 3.2.3. Building Mathematics Model to Extract Parameters Based on Harmonic Fitting and Recursive Total Least Squares

Firstly, format trigonometric functions by the lengths of every period and do LSQ for all cycles; then build the models fit to 12 harmonics. The model can match all the signals with different period cycles in one sample precisely. Because we built the models based on multicycle signals, *T*_*i*_ of each cycle can be dissimilar.

When the values of *y*_*t*_ = *a*_0_ + ∑_*k*=1_^*p*^(*a*_*k*_cos⁡2*ktπ*/*L* + *b*_*k*_sin⁡2*ktπ*/*L*) arrive minimum, the difference of models and the original signals also be the minimum. And *a*_*k*_ and *b*_*k*_ can carry the information of all the cycles. For any cycle whose period length is *L*, we can use

(1) $$\begin{array}{c} y_{t}=a_{0}+\overset{p}{\underset{k=1}{\sum }}\left(\;a_{k}\cos\frac{2kt\pi }{L}+b_{k}\sin\frac{2kt\pi }{L}\;\right) \end{array}$$

to be the model of pulse waves, so *a*_*k*_ and *b*_*k*_ are the parameters we need, and the formula is

(2) ak=∑i=1nTiTai,k,bk=∑i=1nTiTbi,k.

**Figure 5:**
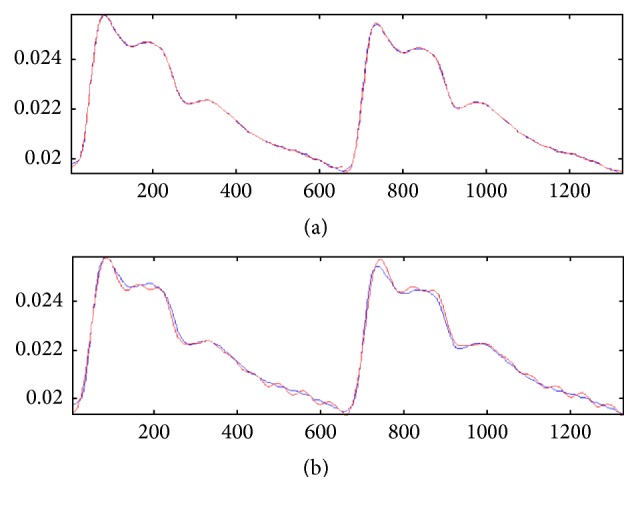
Reduction of error by recursive total least squares: the red signals are original signals and the blue ones are fitting signals. The upper figure shows the fitting effect of the two cycles by separate fitting, and the lower one shows the effect by normal fit. The separate fitting can get a better result.

**Figure 6:**
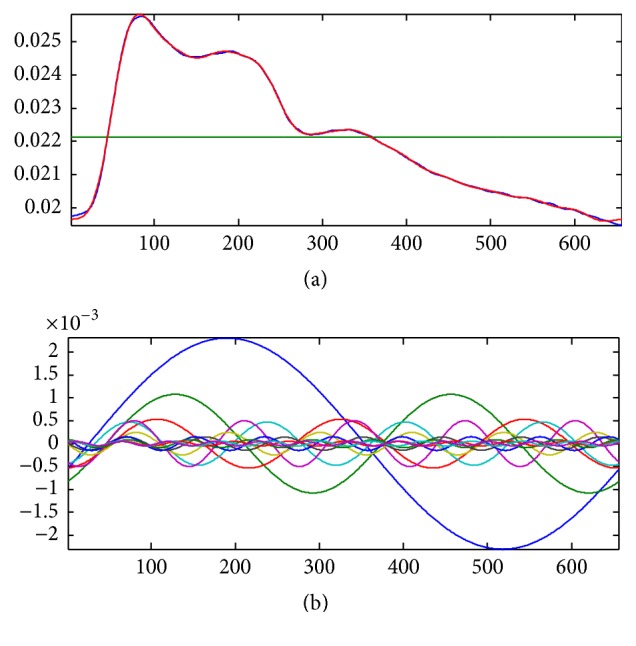
Harmonic fitting: the upper figure shows the signal of one cycle; the red one is the original signal and the blue one is the fitting result by the 12 harmonics shown in the lower figure. The fitting effect is optimal.

In fact we cannot construct a figure of a cycle even if we have all the characters, but we can use *a*_*k*_ and *b*_*k*_ to construct one by the formula (the fitting result is shown in Figures 5 and 6).

#### 3.2.4. Parameters Extracting

**Figure 7:**
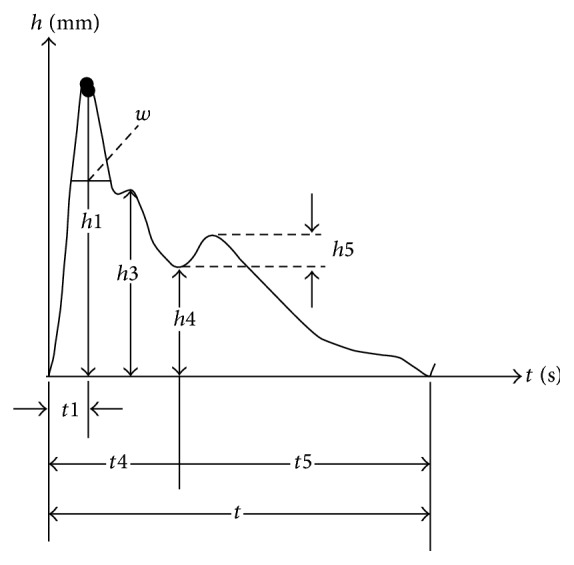
Time domain parameters.

To build models for the pulse waves collected from 6 places (3 places in radial artery of each side) called “cun,” “guan,” and “chi” in TCM and account for the amplitude and phase of 12 harmonics (*C*1–*C*12,* F*1–*F*12), 9 time domain parameters [13] were added such as *h*1, *t*1, *h*4/*h*1, *h*5/*h*1, and *w* (Figure 7 shows the details). Because the period of waves from each place is similar, we can get 193 parameters from every volunteer: 32 parameters × 6 places + 1.

### 3.3. Classification, Identification, and Features Mining

#### 3.3.1. Principal Component Analysis (PCA)

**Figure 8:**
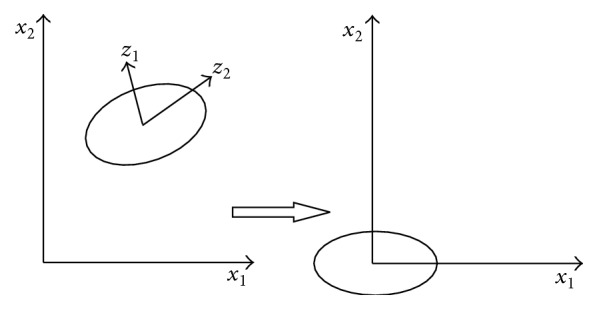
Diagram of data decomposition in PCA.

Principal Component Analysis [14, 15] is a method of multivariate statistical analysis to show the reason of variance of data by linear combination of the parameters. In this study we have extracted 193 parameters from pulse diagnosis signals collected from the 6 places and decomposed all the data to different orthogonal components by PCA, which means reducing the high-dimensional data to several orthogonal independent one-dimensional arrays (Figure 8 shows the details). The first principal component reflects the greatest change direction.

#### 3.3.2. Least Squares Regression (LS)

The purpose of classification and identifications is to establish a method to distinguish two or more groups of known data, and this method can be used to identify some new data. We do it as follows:

(a) Design a one-dimensional vector as the optimal regressand in the meaning of canonical correlation and then do Least squares Regression:
  (3) $$\begin{array}{c} \text{the regressand is }Y={\left[\;\underset{n_{1}}{\underset{︸}{n_{2},\ldots ,n_{2}}},\underset{n_{2}}{\underset{︸}{-n_{1},\ldots ,-n_{1}}}\;\right]}^{T}. \end{array}$$
(b) Subtract the mean of regressors consisting of the extracted parameters in Section 3.2.
(c) Select fewer regressors from the 193 parameters by Extended Forward Backward Least Square Regression (EFBLS) [4].
(d) For constructing the histogram of predicted indices by the regression model, we classify the samples by their indices. According to the histogram, we obtain *p* values of the samples belonging to either group.

#### 3.3.3. Least Absolute Shrinkage and Selection Operator (LASSO)

**Figure 9:**
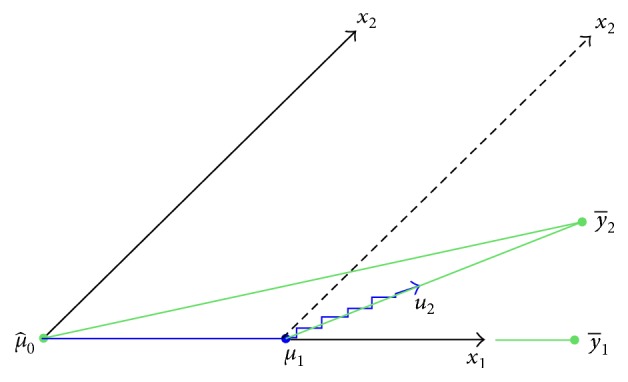
Schematic diagram of LAR: the LAR direction *u*_2_ makes an equal angle with *x*_1_ and *x*_2_.

LASSO [16] is a new variable-choosing method created by Tibshirani in 1996 [17] on the basis of Bridge Regression by Frank and Friedman [18] and Nonnegative Garrote by Breiman [19]. The algorithm is summarized as follows. Suppose *β* is the coefficient of the model, the corresponding function is *l*(*β*), and *β* is a *d*-dimensional vector. The equation of parameters penalty is. When *l*(*β*) = (*y* − *Xβ*)^2^, *p*_*λ*_*i*__(|*β*_*i*_|) = *λ*|*β*_*i*_|^*q*^, that is, the Bridge Regression. When *q* = 1, that is the LASSO (Figure 9 shows the details). In this study, we use LASSO with cross-validation to choose the regressors. It usually selects fewer numbers of the regressors and trade-offs between bias and mean squared error. So it may increase the accuracy of the model for coming new data.

#### 3.3.4. Comprehensive Comparison and Analysis

We compare the results by the 3 methods. According to the result of PCA, the unsupervised learning, we can make sure if there is innate difference between the groups. The result of supervised learning will help the features selection to mine the most important features during the classification. Based on the cross-validation of 3 methods in data analysis, a reliable conclusion can be given in pulse diagnosis signals analysis.

## 4. Results

We have used 163 samples in this research (98 healthy volunteers, 38 patients with FLD, and 27 patients with cirrhosis). The results were based on the 193 parameters extracted from the signals in 3 places of each side of the radial artery called “cun,” “guan,” and “chi” in TCM by PCA, LS, and LASSO.

### 4.1. Signal Analysis between Healthy Volunteers and Patients with FLD

#### 4.1.1. Principal Component Analysis (PCA)

**Figure 10:**
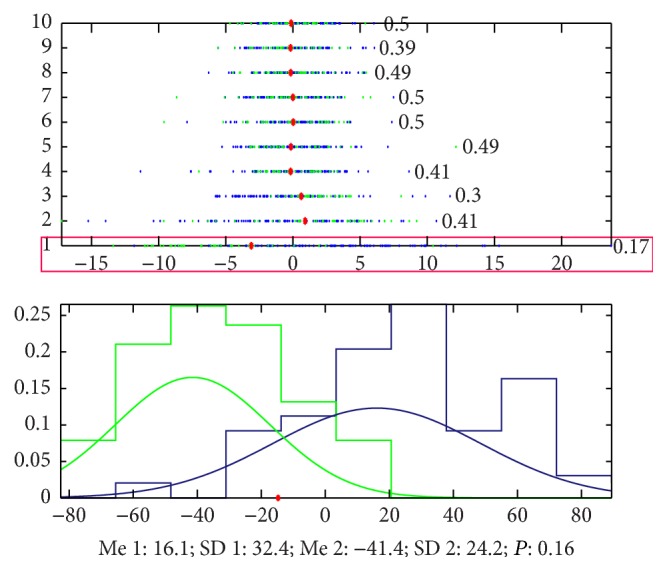
Classification by PCA. The accuracy that we cannot classify the signals of the 2 groups by the 1st principal component is 0.17, which means that the accuracy to classify this 2 groups is 83%. The blue points (patients with FLD) mainly appeared in the left side of the red point and the green ones (healthy volunteers) appeared in the right side. And the accuracy by 1st principal component and the 3rd principal component is 84%.

By using the unsupervised learning without any information to guide the classification, we found that there is obvious difference between the pulse waves of healthy volunteers and patients with FLD. The accuracy to classify the signals of the two groups only by the 1st principal component is 83% (Figure 10 shows the details). The result suggests that it is feasible to separate these two groups without any supervising due to physiological changes.

#### 4.1.2. Supervised Learning: LS and LASSO

According to the character of clinical data, small samples with large dimensionality, we built a program to avoid the false classification in LS method: we test our method by 23 simulated samples to decide the upper limit number of selected regressors according to the number of samples. In this study, we can use 7 regressors at most.

**Figure 11:**
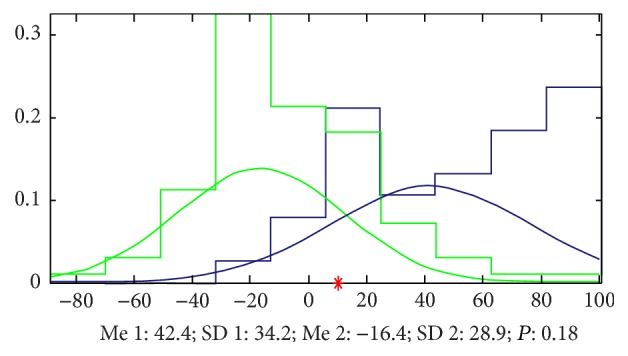
Classification by LS. The accuracy that we cannot classify the signals of the 2 groups by only 2 parameters is 0.18, which means that the accuracy to classify this 2 groups is 82%, and the green samples (healthy volunteers) mainly appeared in the left side of the red point and the blue ones (patients with FLD) appeared in the right side.

*(a) Results by LS*. Doing the analysis by LS, we found that it is very easy to classify the two groups of signals and the accuracy is 91% by 7 parameters and 82% by only 2 parameters. The most important parameters we mined by a method we built named EFBLS (Extended Forward Backward Least Square Regression) mainly appeared at zuocun, youguan, and youchi (Figure 11 and Table 1 show the details).

**Figure 12:**
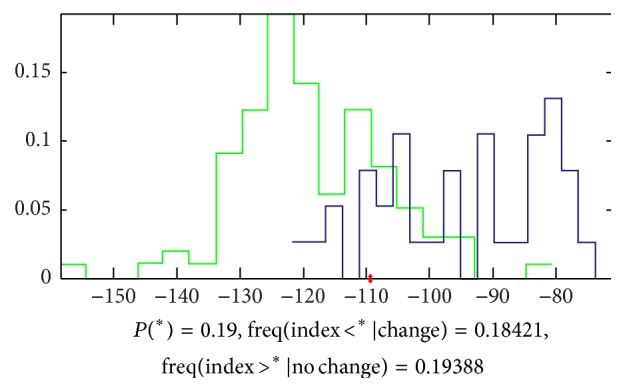
Classification by LASSO. The accuracy that we cannot classify the signals of the 2 groups by 3 parameters is 0.19, which means that the accuracy to classify this 2 groups is 81%, and the green samples (healthy volunteers) mainly appeared in the left side of the red point and the blue ones (patients with FLD) appeared in the right side.

*(b) Results by LASSO*. Doing the analysis by LASSO, we found that there is obvious difference between the two groups of signals. The accuracy of classification is 81% by 3 parameters, and the equation of the model is as follows:

(4) Y=−0.01C2  zuocun−0.11t4  zuochi−0.19F2  youchi

(Figure 12 shows the details).

#### 4.1.3. Comprehensive Comparison and Analysis

Comparing the results from the 3 methods, based on the combination of unsupervised learning and supervised learning, we can make a conclusion that there is obvious difference between the pulse signals of healthy volunteers and FLD patients, and the accuracy of classification is about 85%. The features we mined were mainly focused on zuocun, youguan, and youchi. In the theory of pulse diagnosis of TCM, youguan always represents the function of digestive. If some problem happened on digestive, doctors can feel the pulse in youguan changed. In this study, the result was partly matched with the theory of TCM. However, we need more data to confirm it.

### 4.2. Signal Analysis between Healthy Volunteers and Patients with Cirrhosis

#### 4.2.1. Principal Component Analysis (PCA)

**Figure 13:**
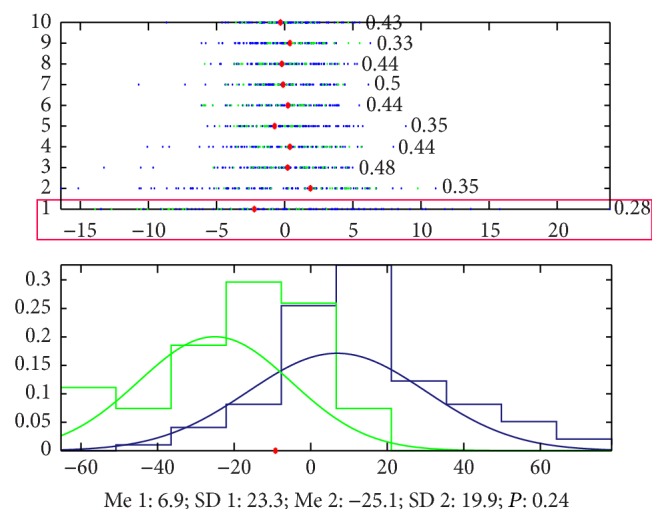
Classification by PCA. The accuracy that we cannot classify the signals of the 2 groups by the 1st principal component is 0.28, which means that the accuracy to classify this 2 groups is 72%, and the blue points (patients with cirrhosis) mainly appeared in the left side of the red point and the green ones (healthy volunteers) appeared in the right side. And the accuracy by 1st principal component and the 9th principal component is 76%.

By using the unsupervised learning without any information to guide the classification, we found that there is obvious difference between the pulse waves of healthy volunteers and patients with cirrhosis. The accuracy to classify the signals of the two groups only by the 1st principal component is 72% (Figure 13 shows the details).

#### 4.2.2. Supervised Learning: LS and LASSO

In this study, we can use up to 7 regressors.

**Figure 14:**
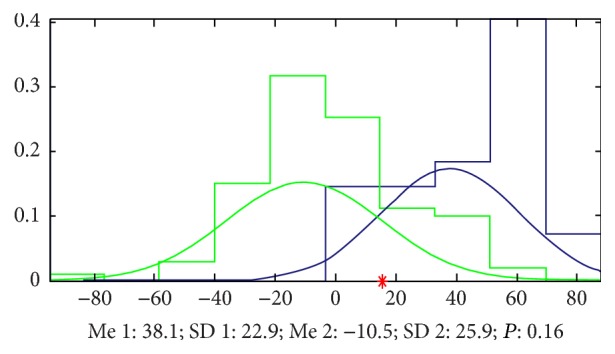
Classification by LS. The accuracy that we cannot classify the signals of the 2 groups by only 2 parameters is 0.16, which means that the accuracy to classify this 2 groups is 84%, and the green samples (healthy volunteers) mainly appeared in the left side of the red point and the blue ones (patients with cirrhosis) appeared in the right side.

*(a) Results by LS*. Doing the analysis by LS, we found that it is very easy to classify the two groups of signals and the accuracy is 93% by 7 parameters and 84% by only 2 parameters. The most important parameters we mined by a method we built named EFBLS (Extended Forward Backward Least Square Regression) mainly appeared at zuoguan, zuochi, and youguan (Figure 14 and Table 2 show the details).

**Figure 15:**
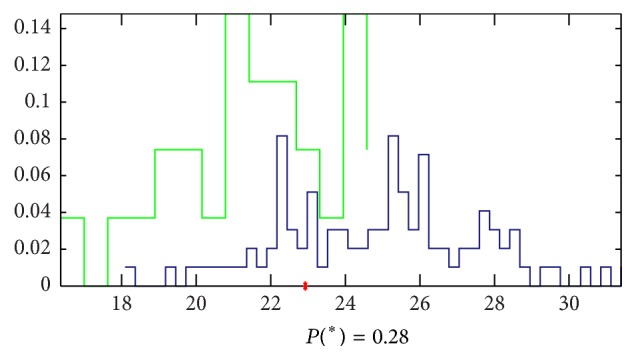
Classification by LASSO. The accuracy that we cannot classify the signals of the 2 groups by 2 parameters is 0.28, which means that the accuracy to classify this 2 groups is 72%, and the green samples (healthy volunteers) mainly appeared in the left side of the red point and the blue ones (patients with cirrhosis) appeared in the right side.

*(b) Results by LASSO*. Doing the analysis by LASSO, we found that there is obvious difference between the two groups of signals. The accuracy to classify is 72% by 2 parameters and the equation of the model is as follows:

(5) $$\begin{array}{c} Y=-0.05C2\text{ youguan}-0.011F1\text{ youchi} \end{array}$$

(Figure 15 shows the details).

#### 4.2.3. Comprehensive Comparison and Analysis

Comparing the results from the 3 methods, based on the combination of unsupervised learning and supervised learning, we can make a conclusion that there are differences between the pulse signals of healthy volunteers and cirrhosis patients, and the accuracy of classification is about 75%. The features we mined were mainly focused on zuoguan. In the theory of pulse diagnosis of TCM, zuoguan always represents the function of liver and gallbladder. If some problem happened on liver and gallbladder, doctors can feel the pulse in youguan changed. In this study, the result was matched with the theory of TCM.

### 4.3. Signal Analysis between Patients with FLD and Cirrhosis

#### 4.3.1. Principal Component Analysis (PCA)

**Figure 16:**
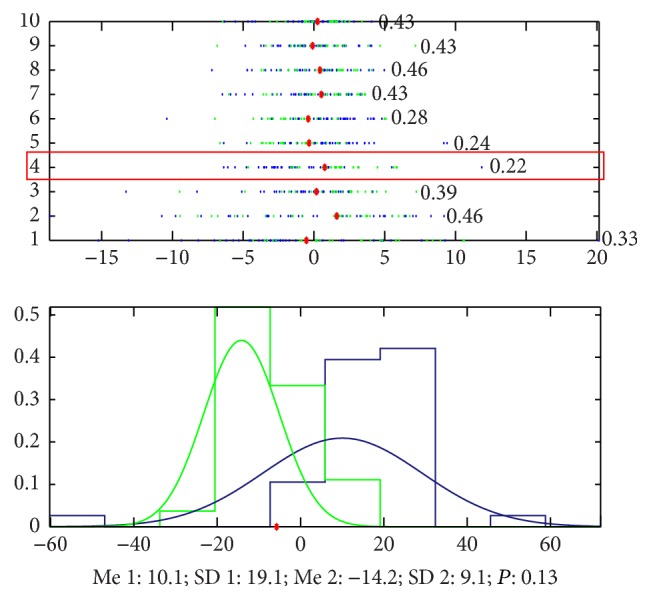
Classification by PCA. The accuracy that we cannot classify the signals of the 2 groups by the 1st principal component is 0.22, which means that the accuracy to classify this 2 groups is 78%, and the blue points (patients with cirrhosis) mainly appeared in the left side of the red point and the green ones (patients with FLD) appeared in the right side. And the accuracy by the 4th principal component and the 5th principal component is 87%.

By using the unsupervised learning without any information to guide the classification, we found that there is obvious difference between the pulse waves of healthy volunteers and patients with cirrhosis. The accuracy to classify the signals of the two groups only by the 4th principal component is 78% (Figure 16 shows the details).

#### 4.3.2. Supervised Learning: LS and LASSO

In this study, we can use up to 7 regressors.

**Figure 17:**
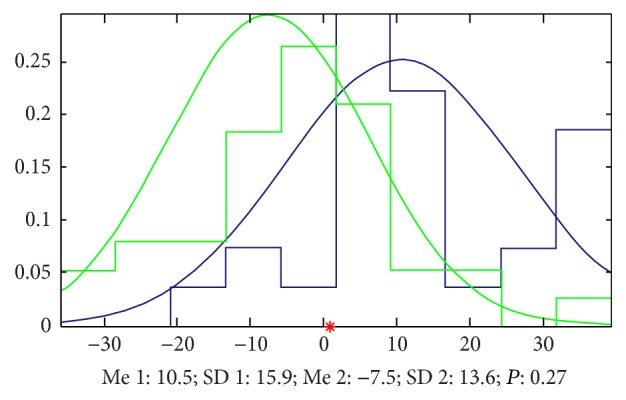
Classification by LS. The accuracy that we cannot classify the signals of the 2 groups by only 2 parameters is 0.27, which means that the accuracy to classify this 2 groups is 73%, and the green samples (patients with FLD) mainly appeared in the left side of the red point and the blue ones (patients with cirrhosis) appeared in the right side.

*(a) Results by LS.* Doing the analysis by LS, we found that it is very easy to classify the two groups of signals and the accuracy is 91% by 7 parameters and 73% by only 2 parameters. The most important parameters we mined by a method we built named EFBLS (Extended Forward Backward Least Square Regression) mainly appeared at zuocun, youguan, and youchi (Figure 17 and Table 3 show the details).

**Figure 18:**
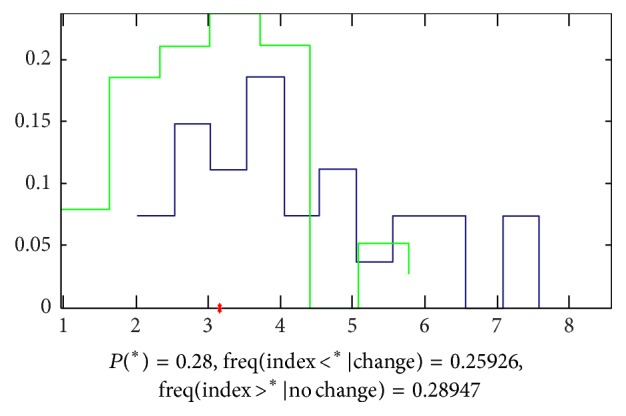
Classification by LASSO. The accuracy that we cannot classify the signals of the 2 groups by 2 parameters is 0.28, which means that the accuracy to classify this 2 groups is 72%, and the green samples (patients with FLD) mainly appeared in the left side of the red point and the blue ones (patients with cirrhosis) appeared in the right side.

*(b) Results by LASSO*. Doing the analysis by LASSO, we found that there is obvious difference between the two groups of signals. The accuracy to classify is 72% by 3 parameters and the equation of the model is as follows:

(6) Y=0.02C1  youchi−0.025C5  youchi−0.05F8  youchi

(Figure 18 shows the details).

#### 4.3.3. Comprehensive Comparison and Analysis

Comparing the results from the cross-validation of the 3 methods, we can make a conclusion that there are differences between the pulse signals of FLD patients and cirrhosis patients, and the accuracy of classification is about 70%. The features we mined were mainly focused on youguan and youchi. This result is not simply matched with the theory of TCM mentioned in Section 4.2.3; zuoguan always represents the function of liver and gallbladder. As we know that FLD and cirrhosis are two liver diseases in different stage and the pathologic changes are much more serious in not only liver but also blood vessels and digestive system. So the features appear on other point instead of zuoguan.

## 5. Conclusions

There is a significant difference between the pulse diagnosis signals of healthy volunteers and patients with FLD and cirrhosis, and the result was confirmed by 3 analysis methods. The identification accuracy of the 1st principal component is about 75% without any classification formation by PCA, and supervised learning's accuracy (LS and LASSO) was even more than 93% when 7 parameters were used and 84% when only 2 parameters were used. From the results, we can have some conclusions.

(1) The machine learning method we built based on the combination of unsupervised learning, PCA, and supervised learning, LS and LASSO, is feasible in analyzing the pulse diagnosis signals. Moreover, according to the result of cross-reference by 3 methods and the equation established by LASSO, we can achieve a reliable result by signals of pulse diagnosis in TCM to identify the healthy volunteers and the patients. This method can help offer some objective data to prove the important role of pulse diagnosis in TCM.

(2) The features we mined by LS and LASSO to classify the healthy volunteers and patients with FLD and cirrhosis appear in specific places we called “cun,” “guan,” and “chi” when feeling the pulse. For example, the features to classify the healthy volunteers and patients with FLD mainly appear in zuocun, youguan, and youchi. The features to classify the healthy volunteers and patients with cirrhosis mainly appear in zuoguan and zuochi. However, youguan and youchi are the main place where features appear between the patients of FLD and cirrhosis patients. This result is similar to the theory of pulse diagnosis in Traditional Chinese Medicine (TCM) which can support the modern research of pulse diagnosis. But more research is needed to confirm the conclusion.

(3) FLD and cirrhosis are the most common liver diseases, and there are high incidences among middle-aged men. These two kinds of diseases have not only common points in the pathology but also different points. Pulse diagnosis of TCM can diagnose disease by feeling the pulse of radial artery, but we have no evidence to prove that. In this study, according to the result, we can find that from the pulse wave collected in different places called “cun,” “guan,” and “chi” in radial artery humans in different health condition can be classified with high accuracy even when the diseases affected the same organ, liver. From the results, we can offer some important evidence for the science of pulse diagnosis in TCM clinical diagnosis. Of course, we need more data to confirm it.

In a word, the machine learning method we built in this study based on the combination of unsupervised learning, PCA, and supervised learning, LS and LASSO, might offer some confidence for the realization of computer-aided diagnosis by pulse diagnosis in TCM. In addition, this study might offer some important evidence for the science of pulse diagnosis in TCM clinical diagnosis.

## Figures and Tables

**Table 1:** Main parameters used in the LS model.

Main parameters	Parameters		
	*C*10 youguan	*C*2 youchi	*C*2 zuocun
Used times	20	15	14
Coefficient (importance)	1.12	1.35	1.28
Mean	−516.72	1412.28	1102.34
SD	55.10	162.67	98.03

**Table 2:** Main parameters used in the LS model.

Main parameters	Parameters		
	*C*1 zuoguan	*C*2 zuoguan	*C*9 zuochi
Used times	20	20	20
Coefficient (importance)	1.29	1.35	1.11
Mean	−1456.89	1417.54	647.32
SD	74.20	87.70	44.19

**Table 3:** Main parameters used in the LS model.

Main parameters	Parameters				
	*C*3 youguan	*C*1 youchi	*C*4 youchi	*F*1 youchi	*C*9 zuocun
Used times	20	20	18	18	18
Coefficient (importance)	1.67	1.67	1.31	1.32	1.23
Mean	1218.55	−965.47	−708.04	2.42	366.40
SD	97.44	88.77	99.54	0.26	30.25
